# Supplementary material for: Towards an Evidence-Based Classification System for Para Dressage: Associations between Impairment and Performance Measures
Source: Animals (Basel). 2023 Aug 31;13(17):2785. doi: 10.3390/ani13172785 (PMC10487214; doi:10.3390/ani13172785)
Supplement: Supplementary file 1 [file animals-13-02785-s001.zip › File S1.pdf]

Supplementary Tables

Descriptive statistics for Para athletes separated by grade for impairment and performance measurements.

Table S1. Average scores for Para athletes by grade and non-disabled athletes by level. Function In Sitting Test (FIST), Scale and Assessment of Rating for Ataxia (SARA), Trunk Impairment Scale (TIS), and Re-Modified Ashworth Scale (R-MAS). Peak handheld dynamometry measures (N) for the trunk are presented. Note: %COV for SARA and R-MAS were calculated by dividing the SD by the difference between the mean score and total available score. Grand Prix (GP), Prix St. Georges (PSG).

						Trunk Handheld Dynamometry (peak values)					
	n	FIST	SARA	TIS	R-MAS	Flexion	Extension	Rotation to Left	Rotation to Right	Left Lateral Flexion	Right Lateral Flexion
Grade											
1	6	41.17 (12.38)	7.75 (3.33)	7.00 (5.18)	6.17 (6.49)	63.8 (38.2)	80.9 (44.6)	37.6 (22.6)	37.1 (31.0)	48.9 (25.7)	51.3 (18.8)
%COV		30	27	74	36	60	55	60	84	53	37
2	3	48.00 (7.55)	4.33 (1.53)	9.67 (6.43)	5.33 (4.73)	46.5 (20.7)	52.8 (13.4)	42.8 (16.6)	42.3 (16.0)	58.6 (26.3)	47.7 (13.2)
%COV		16	10	67	25	45	25	39	38	45	28
3	4	48.25 (3.30)	4.63 (3.20)	15.00 (7.48)	8.75 (11.12)	68.2 (39.9)	71.1 (57.9)	54.7 (26.1)	59.9 (24.5)	70.2 (41.8)	61.3 (40.5)
%COV		7	21	50	73	59	81	48	41	60	66
4	6	55.50 (1.22)	3.42 (3.65)	18.17 (3.60)	2.33 (4.08)	64.2 (33.7)	86.5 (49.4)	69.1 28.9)	62.0 (27.9)	83.2 (33.5)	76.3 (32.4)
%COV		2	22	20	19	52	57	42	45	40	42
5	2	56.00 (0.00)	0.50 (0.71)	21.00 (2.83)	1.00 (1.41)	48.4 (1.6)	67.9 (12.8)	73.3 (8.8)	64.0 (24.0)	72.1 (4.5)	71.8 (5.3)
%COV		0	4	13	6	3	19	12	38	6	7
Level											
GP	5	56.00 (0.00)	0.0 (0.0)	19.60 (0.89)	0.00 (0.00)	108.5 (26.3)	157.4 (43.5)	88.0 (14.2)	88.5 (21.5)	116.3 (26.9)	118.3 (21.0)
%COV		0	0	4	0	24	28	16	24	23	18
PSG	6	56.00 (0.00)	0.0 (0.0)	20.83 (1.60)	0.33 (0.82)	103.1 (18.8)	128.8 (40.9)	93.0 (25.0)	86.0 (20.8)	125.3 (34.4)	118.9 (27.7)
%COV		0	0	8	4	18	32	27	24	27	23

Table S2. Average scores for Para athletes by grade and non-disabled athletes by level for peak handheld dynamometry (HHD) measures (N) for the limbs. Adduction (Add), External Rotation (Ex Rot), Extension (Extn). Grand Prix (GP), Prix St. Georges (PSG).

		Hip (peak values)				Shoulder (peak values)				Elbow (peak values)			
	n	Add Right	Add Left	Ex Rot Right	Ex Rot Left	Extn Right	Extn Left	Ex Rot Right	Ex Rot Left	Flexio n Right	Flexio n Left	Ex Rot Right	Ex Rot Left
Grade													
1	6	43.1 (78.4)	40.9 (70.9)	17.83 (32.17)	17.82 (22.10)	47.9 (25.5)	45.9 (32.9)	27.7 (15.5)	36.0 (24.6)	44.2 (38.1)	40.7 (48.5)	37.6 (29.1)	39.7 (37.6)

%COV		182	173	180	124	53	72	56	68	86	119	77	95
2	3	29.8 (28.5)	27.9 (24.8)	19.83 (17.92)	23.85 (23.51)	49.0 (21.9)	54.8 (28.3)	42.0 (12.8)	52.0 (19.3)	46.7 (19.8)	42.7 (12.9)	56.4 (25.7)	62.0 (36.1)
%COV		96	89	90	99	45	52	30	37	42	30	46	58
3	4	62.1 (69.3)	53.9 (67.4)	18.04 (21.47)	18.81 (22.71)	73.5 (56.0)	73.9 (43.2)	54.5 (32.0)	64.4 (37.7)	66.4 (35.1)	61.3 (33.6)	79.4 (58.9)	81.8 (57.8)
%COV		112	125	119	121	76	58	59	59	53	55	74	71
4	6	63.3 (31.9)	59.4 (24.5)	53.01 (25.08)	54.54 (28.03)	72.4 (49.3)	71.3 (40.0)	53.0 (15.5)	53.8 (21.0)	55.3 (30.3)	65.4 (32.2)	49.3 (18.4)	59.0 (24.5)
%COV		50	41	47	51	68	56	29	39	55	49	37	42
5	2	83.3 (31.9)	61.0 (40.9)	54.13 (21.67)	48.43 (32.63)	47.7 (4.3)	63.3 (17.0)	43.1 (5.5)	46.3 (0.9)	52.3 (3.6)	51.7 (3.2)	43.2 (4.2)	56.0 (2.3)
%COV		38	67	40	67	9	27	13	2	7	6	10	4
Level													
GP	5	128.1 (29.9)	120.9 (26.0)	79.7 (19.6)	86.9 (32.2)	118.5 (30.5)	122.0 (27.7)	96.5 (25.3)	96.8 (21.2)	99.8 (21.0)	110.2 (34.1)	99.9 (24.4)	113.6 (25.6)
%COV		23	22	25	37	26	23	26	22	21	31	24	23
PSG	6	112.3 (32.6)	114.6 (41.2)	74.4 (18.3)	72.6 (17.2)	109.0 (30.2)	100.2 (32.0)	76.0 (18.8)	82.0 (18.4)	90.4 (30.3)	93.5 (22.2)	83.1 (19.3)	91.6 (23.5)
%COV		29	36	25	24	28	32	25	22	34	24	23	26

Table S3. Average scores for Para athletes by grade and non-disabled athletes by level for performance measures. These are as follows; 3D simulator trunk rotation signal power (SPowersf) ($deg^2.s$) and harmonic ratio (SRatiosf) at the stride frequency during simulated walk and trot, head stability (%), dynamic symmetry and symmetry vector for the trunk (Sym Trunk, SVector Trunk) and pelvis (Sym Pelvis, SVector Pelvis) (normalised to trunk length and pelvis width respectively) during walk, average absolute deviation of detrended coordination variability of 3D trunk to pelvis rotation (deg) during simulated walk (DVar walk) and trot (DVar Trot). Grand Prix (GP), Prix St. Georges (PSG).

	n	$SPower_{sf}$ walk	$SRatio_{sf}$ walk	$SPower_{sf}$ trot	$SRatio_{sf}$ trot	Head Stability	Sym Trunk	SVector Trunk	Sym Pelvis	SVector Pelvis	DVar walk	DVar trot
Grade												
1	6	1.25 (0.07)	1.36 (0.06)	0.39 (0.37)	7.23 (9.45)	-29.7 (28.1)	1.99 (1.71)	0.22 (0.24)	1.14 (0.54)	0.20 (0.05)	17.63 (13.84)	8.47 (10.86)
%COV		6	4	95	131	95	86	109	47	25	79	128
2	3	1.24 (0.10)	1.36 (0.01)	0.16 (0.03)	3.04 (0.91)	-7.0 (25.5)	1.92 (0.86)	0.17 (0.16)	1.12 (0.33)	0.11 (0.03)	11.96 (3.32)	5.48 (0.30)
%COV		8	1	19	30	364	45	94	29	27	28	5
3	4	1.20 (0.25)	1.42 (0.10)	0.18 (0.03)	2.97 (1.18)	-16.8 (22.5)	1.69 (0.58)	0.17 (0.13)	1.90 (1.37)	0.20 (0.04)	13.09 (4.39)	4.57 (0.67)
%COV		21	7	17	40	134	34	76	72	20	34	15
4	6	1.34 (0.03)	1.41 (0.13)	0.19 (0.03)	3.15 (1.39)	-2.4 (14.0)	2.04 (0.49)	0.11 (0.06)	1.28 (0.44)	0.17 (0.04)	11.63 (4.19)	10.50 (15.59)
%COV		2	9	16	44	583	24	55	34	24	36	148

5	2	1.33	1.35	0.19	2.89	-5.3	1.35	0.18	0.79	0.17	6.45	4.18
		(0.04)	(0.02)	(0.003)	(0.64)	(9.1)	(0.21)	(0.01)	(0.24)	(0.02)	(1.00)	(0.64)
%COV		3	1	2	22	172	16	6	30	12	16	15
Level												
GP	5	0.74	1.38	0.17	2.76	-3.1	1.11	0.11	1.07	0.18	9.33	4.92
		(0.43)	(0.05)	(0.05)	(0.41)	(13.7)	(0.24)	(0.05)	(0.14)	(0.02)	(2.79)	(2.18)
%COV		58	4	29	15	442	22	45	13	11	30	44
PSG	6	1.32	1.33	0.22	9.46	9.4	0.75	0.06	0.98	0.18	8.14	6.46
		(0.05)	(0.04)	(0.03)	(15.7)	(12.8)	(0.40)	(0.04)	(0.24)	(0.03)	(3.06)	(1.74)
%COV		4	3	14	166	136	53	66	24	17	38	27
